# Supplementary material for: Mitochondrial DNA Variants in Patients with Liver Injury Due to Anti-Tuberculosis Drugs
Source: J Clin Med. 2019 Aug 13;8(8):1207. doi: 10.3390/jcm8081207 (PMC6723168; doi:10.3390/jcm8081207)
Supplement: Supplementary file 1 [file jcm-08-01207-s001.pdf]

Supplementary File

Sequencing of the entire mitochondrial genome by using NGS

The methodology captures sequencing information during replication of the target DNA [1,2]. The template was closed, single-stranded circular DNA created by ligating hairpin adaptors to both ends of the target double-stranded DNA. During the sequencing process, the template diffused into a sequencing unit that contains a single polymerase of a smallest volume. Upon addition of four fluorescent-labeled nucleotides that generate distinct spectra, the polymerase within each unit incorporated the nucleotides and fluorescent signals were emitted. The signals were recorded using a camera in real-time. As the replication processes of the target DNA continued, both strands of the target DNA could be sequenced multiple times.

After the system had generated the raw sequence data, a circular consensus sequence read was performed using PacBio's instrument control and SMRT Analysis software (http://www.pacificbiosciences.com). The read quality was checked using a Perl script (http://david.abcc.ncifcrf.gov/manuscripts/pacbio_qc) to produce a summary read-QC-parameter report. High-quality circular consensus sequence reads were aligned to the standard revised Cambridge Reference Sequence (NC_012920.1, *Homo sapiens* mitochondrion complete genome), and variants were called and annotated with dbSNP IDs (http://www.ncbi.nlm.nih.gov/SNP/) by using SMRT Analysis software. The definition of the variants was the positions with nucleotide different from that in the reference sequence. Variants were defined as novel if they were not present in the MitoMAP database (updated March 19, 2018). The effects of nonsynonymous variants on protein function were predicted using the scale-invariant feature transform algorithm.

N-acetyltransferase 2 (*NAT2*) genotype determination

NAT2 genotype was determined from genomic DNA by using direct sequencing in 34 patients with and 38 patients without DILI. Of 24 polymorphic sites of *NAT2* gene, four lead to amino acid changes: 191G>A (R64Q), 341T>C (I114T), 590G>A (R197Q) and 857G>A (G286E). Presence of two of these variant alleles, one variant allele plus one common allele, and two common alleles in a patient was defined as slow, intermediate, and rapid acetylator genotypes, respectively [3,4].

References

1. Rhoads, A.; Au, K.F. Pacbio sequencing and its applications. *Genomics Proteomics Bioinformatics* **2015**, *13*, 278-289.
2. Ardui, S.; Ameur, A.; Vermeesch, J.R.; Hestand, M.S. Single molecule real-time (smrt) sequencing comes of age: Applications and utilities for medical diagnostics. *Nucleic Acids Res* **2018**, *46*, 2159-2168.
3. Hein, D.W.; Doll, M.A. Accuracy of various human nat2 snp genotyping panels to infer rapid, intermediate and slow acetylator phenotypes. *Pharmacogenomics* **2012**, *13*, 31-41.
4. Wang, J.Y.; Liu, C.H.; Hu, F.C.; Chang, H.C.; Liu, J.L.; Chen, J.M.; Yu, C.J.; Lee, L.N.; Kao, J.H.; Yang, P.C. Risk factors of hepatitis during anti-tuberculous treatment and implications of hepatitis virus load. *J Infect* **2011**, *62*, 448-455.

Table S1. Concomitant medications in the 38 patients with drug-induced liver injury during anti-TB treatment (A) and the 38 without (B).

S1A

Concomitant medication	No. (%)
Potential hepatotoxic drug*	
Irbesartan	2 (5%)
Metformin	2 (5%)
Fluvastatin	1 (3%)
Leflunomide	1 (3%)
Sulfasalazine	1 (3%)
Trichlormethiazide	1 (3%)
Non-hepatotoxic drug	
Salmeterol/Fluticasone, inhaled	4 (11%)
Amlodipine	4 (11%)
Isosorbide mononitrate	3 (8%)
Furosemide	3 (8%)
Salbutamol, inhaled	2 (5%)
Tiotropium, inhaled	2 (5%)
Prednisolone	2 (5%)
Coumadin	2 (5%)
Aminophylline	2 (5%)
Doxazosin	2 (5%)
Aspirin	2 (5%)
Digoxin	2 (5%)
Hydroxychloroquine	2 (5%)
Glimepiride	2 (5%)
Zoledronic	1 (3%)
Ceftriaxone, intravenous	1 (3%)
Carvedilol	1 (3%)
Diltiazem	1 (3%)
Bumetanide	1 (3%)
Ipratropium, inhaled	1 (3%)
Mesna	1 (3%)
Celecoxib	1 (3%)
Buflomedil	1 (3%)
Piperacillin/Tazobactam, intravenous	1 (3%)
Letrozole	1 (3%)
Colchicine	1 (3%)

* A total of 5 (13%) patients received potential hepatotoxic drugs during anti-TB treatment. Among them, one received irbesartan, sulfasalazine and leflunomide concomitantly, and another received metformin and fluvastatin concomitantly.

S1B

Concomitant medication	No. (%)
Potential hepatotoxic drug*	
Metformin	4 (11%)
Tegafur/Uracil	2 (5%)
Tacrolimus	2 (5%)
Mycophenolate mofetil	2 (5%)

Irbesartan	1 (3%)
Acarbose	1 (3%)
Atorvastatin	1 (3%)
Trichlormethiazide	1 (3%)
Non-hepatotoxic drug	
Glimepiride	5 (13%)
Prednisolone	3 (8%)
Amlodipine	3 (8%)
Aspirin	3 (8%)
Medroxyprogesterone	2 (5%)
Betaxolol	1 (3%)
Digoxin	1 (3%)
Carvedilol	1 (3%)
Efavirenz	1 (3%)
Metoclopramide	1 (3%)
Estazolam	1 (3%)
Phenytoin	1 (3%)
Felodipine	1 (3%)
Sertraline	1 (3%)
Fexofenadine	1 (3%)
Finasteride	1 (3%)
Morphine	1 (3%)
Glibenclamide	1 (3%)
Pentoxifylline	1 (3%)
Tamsulosin	1 (3%)
Pioglitazone	1 (3%)
Ramipril	1 (3%)
Zolpidem	1 (3%)
Clopidogrel	1 (3%)
Losartan	1 (3%)
Alendronic/Colecalciferol	1 (3%)
Terazosin	1 (3%)
Thyroxine	1 (3%)
Indapamide	1 (3%)
Abacavir/Lamivudine	1 (3%)

* A total of 9 (24%) patients received potential hepatotoxic drugs during anti-TB treatment. Among them, one received tegafur/uracil, atorvastatin, acarbose concomitantly, two received mycophenolate mofetil and tacrolimus concomitantly, and another received trichlormethiazide and metformin concomitantly.

Table S2. Thirteen TB patients who succumbed within 2 years of initiation of anti-TB treatment.

No.	Group	Sex	Age (y)*	Co-existing diseases	Death day#	Cause of death	Die of TB
1	DILI (H)	F	43	Nil	22	TB septic shock & respiratory failure	Yes
2	DILI (H)	M	72	Disseminated lung cancer	98	Pneumonia & respiratory failure	No
3	DILI (H)	F	43	Disseminated breast cancer	170	Lung metastases & respiratory failure	No
4	DILI (R)	M	92	COPD, CAD, hepatitis C, CKD	158	COPD, pneumonia & respiratory failure	Yes
5	DILI (R)	M	69	COPD, esophageal cancer, old stroke	281	COPD, pneumonia & respiratory failure	No
6	DILI (R)	F	82	CAD, CHF, Sjogren's syndrome	79	Miliary TB with respiratory failure	Yes
7	DILI (R)	M	50	CAD, CHF, DM	75	Pulmonary TB, CHF & respiratory failure	Yes
8	DILI (R)	M	75	Hepatitis C, hypertension, cachexia	117	Pneumonia, cachexia & respiratory failure	Yes
9	DILI (R)	M	56	Dermatomyositis, APS, under chemotherapy	497	Septic shock	No
10	DILI (Z)	F	77	Valvular heart disease, CHF, diabetes mellitus	38	Septic & cardiogenic shock	Yes
11	Non-DILI	M	52	Severe aplastic anemia	38	Pulmonary TB, CMV pneumonia & respiratory failure	Yes
12	Non-DILI	M	84	Colon cancer, DM, old stroke, hypertension	41	Pulmonary TB with respiratory failure	Yes
13	Non-DILI	F	84	Major depression	240	Sputum impaction	No

Abbreviations: APS, antiphospholipid syndrome; CAD, coronary artery disease; CHF, congestive heart failure; CKD, chronic kidney disease; COPD, chronic obstructive pulmonary disease; DILI, drug-induced liver injury; DM, diabetes mellitus; H, isoniazid; R, rifampin; TB, tuberculosis; Z, pyrazinamide. * mean: 67.6; standard deviation: 16.8. # Duration (days) from initiation of anti-TB treatment to death.

Table S3. mtDNA sequences observed in all of our patients that are different from the revised Cambridge Reference Sequence [12] and represent haplogroup-associated polymorphisms [20].

Nucleotide position	mtDNA region	Sequence in rCRS	Sequence in this cohort
263	D-Loop	A	G (100%)
750	RRNS	A	G (100%)
1438	RRNS	A	G (100%)
2706	RRNL	A	G (100%)
7028	COX1	C	T (100%)
8860	ATP6	A	G (100%)
11719	ND4	G	A (100%)
15326	CYTB	A	G (100%)

Table S4. Positions and numbers of mtDNA variants found in patients with and without drug-induced liver injury (DILI).

	Position in mtDNA	rs number	Gene name	Locus	Allele change	Amino acid change	No. of variants found	
							DILI	Non-DILI
1	52			D-loop	T52C		1	0
2	53			D-loop	G53A		1	0
3	54			D-loop	G54C		1	0
4	93	rs369034419		D-loop	A93G		0	1
5	94			D-loop	G94A		1	0
6	103	rs369070397		D-loop	G103A		0	2
7	131			D-loop	T131C		0	1
8	143	rs375589100		D-loop	G143A		1	1
9	146	rs370482130		D-loop	T146C		8	5
10	150	rs62581312		D-loop	C150T		7	9
11	151			D-loop	C151T		1	0
12	152	rs117135796		D-loop	T152C		6	10
13	153	rs370716192		D-loop	A153G		1	3
14	183	rs113913230		D-loop	A183G		1	3
15	185			D-loop	G185A		1	1
16	188			D-loop	A188G		0	1
17	189	rs371543232		D-loop	A189G		1	1
18	194			D-loop	C194T		1	1
19	195	rs2857291		D-loop	T195C		4	1
20	199	rs72619362		D-loop	T199C		5	5
21	200	rs372099630		D-loop	A200G		1	1
22	204	rs3135032		D-loop	T204C		1	2
23	207	rs369669319		D-loop	G207A		2	4
24	210	rs368534078		D-loop	A210G		0	4
25	215	rs372439069		D-loop	A215G		0	1
26	228	rs41323649		D-loop	G228A		1	0
27	234	rs368463610		D-loop	A234G		1	0
28	235	rs3937037		D-loop	A235G		1	4
29	249			D-loop	A249G		1	0
30	279			D-loop	T279C		0	2
31	298			D-loop	C298T		1	0
32	308			D-loop	C308T		0	1
33	309			D-loop	C309T		12	10
34	310	rs369786048		D-loop	T310C		0	1
35	316			D-loop	G316A		1	0
36	318			D-loop	T318C		1	0
37	385			D-loop	A385G		0	1
38	391			D-loop	T391C		0	1
39	456	rs41356551		D-loop	C456T		0	2
40	471			D-loop	T471C		1	0
41	479			D-loop	A479G		1	0
42	489	rs28625645		D-loop	T489C		19	15
43	499	rs3901846		D-loop	G499A		1	0
44	513			D-loop	G513A		2	0
45	572			D-loop	C572A		1	0
46	663		*RRNS*	12srRNA	A663G		1	4
47	681			12srRNA	T681C		0	2
48	709	rs2853517	*RRNS*	12srRNA	G709A		8	10
49	710	rs28358568	*RRNS*	12srRNA	T710C		0	1
50	747		*RRNS*	12srRNA	A747G		1	0
51	752	rs201831870	*RRNS*	12srRNA	C752T		2	0
52	827	rs28358569	*RRNS*	12srRNA	A827G		1	0
53	930	rs41352944	*RRNS*	12srRNA	G930A		0	2
54	942	rs28579222	*RRNS*	12srRNA	A942G		1	0
55	961	rs3888511	*RRNS*	12srRNA	T961C		2	0
56	1005	rs111033179	*RRNS*	12srRNA	T1005C		2	2
57	1041	rs58327546	*RRNS*	12srRNA	A1041G		1	2
58	1048	rs2000974	*RRNS*	12srRNA	C1048T		0	2

59	1107		*RRNS*	12srRNA	T1107C		2	2
60	1119	rs397515724	*RRNS*	12srRNA	T1119C		2	0
61	1189	rs28358571	*RRNS*	12srRNA	T1189C		0	1
62	1382	rs111033358	*RRNS*	12srRNA	A1382C		1	1
63	1390		*RRNS*	12srRNA	A1390G		0	1
64	1503	rs727503164	*RRNS*	12srRNA	G1503A		0	1
65	1520		*RRNS*	12srRNA	T1520C		1	0
66	1541		*RRNS*	12srRNA	T1541C		1	3
67	1598	rs3135027	*RRNS*	12srRNA	G1598A		0	2
68	1664	rs200807305	*TRNV*	tRNA-Val	G1664A		1	1
69	1709	rs200251800	*RRNL*	16srRNA	G1709A		1	1
70	1719	rs3928305	*RRNL*	16srRNA	G1719A		2	0
71	1736	rs193303007	*RRNL*	16srRNA	A1736G		1	4
72	1809		*RRNL*	16srRNA	T1809C		1	0
73	1811	rs28358576	*RRNL*	16srRNA	A1811G		1	1
74	1824		*RRNL*	16srRNA	T1824C		2	1
75	1888	rs28358577	*RRNL*	16srRNA	G1888A		0	1
76	1978		*RRNL*	16srRNA	A1978G		1	0
77	2010		*RRNL*	16srRNA	T2010C		0	1
78	2220		*RRNL*	16srRNA	A2220G		1	0
79	2226		*RRNL*	16srRNA	T2226C		1	0
80	2399		*RRNL*	16srRNA	A2399G		0	1
81	2831	rs199713564	*RRNL*	16srRNA	G2831A		1	0
82	2835		*RRNL*	16srRNA	C2835T		2	1
83	2882	rs3864199	*RRNL*	16srRNA	T2882C		1	0
84	2887		*RRNL*	16srRNA	T2887C		1	0
85	3010	rs3928306	*RRNL*	16srRNA	G3010A		7	3
86	3144		*RRNL*	16srRNA	A3144G		0	1
87	3206	rs200999343	*RRNL*	16srRNA	C3206T		3	0
88	3290	rs199474665	*TRNL1*	tRNA-Leu	T3290C		1	1
89	3316	rs2853516	*ND1*	ND1	G3316A	Ala→Thr	1	2
90	3394	rs41460449	*ND1*	ND1	T3394C	Tyr→His	1	2
91	3397	rs199476120	*ND1*	ND1	A3397G	Met→Val	1	0
92	3398	rs201212638	*ND1*	ND1	T3398C	Met→Thr	0	1
93	3421		*ND1*	ND1	G3421A	Val→Ile	1	0
94	3434	rs202123618	*ND1*	ND1	A3434G	Tyr→Cys	0	1
95	3435		*ND1*	ND1	C3435T	Syn	1	0
96	3497	rs200319905	*ND1*	ND1	C3497T	Ala→Val	2	0
97	3511		*ND1*	ND1	A3511G	Thr→Ala	1	1
98	3537		*ND1*	ND1	A3537G	Syn	0	3
99	3543		*ND1*	ND1	C3543T	Syn	1	0
100	3552	rs28358587	*ND1*	ND1	T3552A	Syn	0	1
101	3571	rs200453691	*ND1*	ND1	C3571T	Leu→Phe	2	0
102	3606		*ND1*	ND1	A3606G	Syn	3	1
103	3675		*ND1*	ND1	A3675T	Syn	1	0
104	3705		*ND1*	ND1	G3705A	Syn	1	1
105	3714		*ND1*	ND1	A3714G	Syn	1	0
106	3744		*ND1*	ND1	A3744G	Syn	0	2
107	3832		*ND1*	ND1	C3832A	Leu→Met	5	5
108	3852		*ND1*	ND1	C3852T	Syn	0	1
109	3861		*ND1*	ND1	A3861G	Syn	1	0
110	3865		*ND1*	ND1	A3865G	Ile→Val	0	1
111	3873		*ND1*	ND1	A3873G	Syn	1	0
112	3882		*ND1*	ND1	G3882A	Syn	1	0
113	3970		*ND1*	ND1	C3970T	Syn	12	10
114	4047		*ND1*	ND1	T4047C	Syn	0	1
115	4048	rs201629275	*ND1*	ND1	G4048A	Asp→Asn	1	3
116	4071		*ND1*	ND1	C4071T	Syn	5	5
117	4086		*ND1*	ND1	C4086T	Syn	6	1
118	4113	rs374117905	*ND1*	ND1	G4113A	Syn	0	1
119	4129	rs201832206	*ND1*	ND1	A4129G	Thr→Ala	1	0
120	4131		*ND1*	ND1	A4131G	Syn	1	0
121	4140		*ND1*	ND1	C4140T	Syn	2	0
122	4164		*ND1*	ND1	A4164G	Syn	1	3

123	4386		*TRNQ*	tRNA-Gln*	T4386C		0	1
124	4454		*TRNM*	tRNA-Met	T4454C		2	0
125	4491	rs201172504	*ND2*	ND2	G4491A	Val→Ile	1	3
126	4562		*ND2*	ND2	A4562G	Syn	1	0
127	4688		*ND2*	ND2	T4688C	Syn	1	0
128	4715	rs28357976	*ND2*	ND2	A4715G	Syn	2	3
129	4769*	rs3021086	*ND2*	ND2	A4769G	Syn	35	37
130	4811		*ND2*	ND2	A4811G	Syn	1	0
131	4820	rs28357977	*ND2*	ND2	G4820A	Syn	1	0
132	4824		*ND2*	ND2	A4824G	Thr→Ala	1	5
133	4850		*ND2*	ND2	C4850T	Syn	4	2
134	4859		*ND2*	ND2	T4859C	Synoymous	1	0
135	4883		*ND2*	ND2	C4883T	Syn	8	5
136	4895		*ND2*	ND2	A4895G	Syn	0	1
137	4973		*ND2*	ND2	T4973C	Syn	1	0
138	4991		*ND2*	ND2	G4991A	Syn	0	1
139	5054	rs28570593	*ND2*	ND2	G5054C	Syn	1	1
140	5093		*ND2*	ND2	T5093C	Syn	0	2
141	5147	rs367778601	*ND2*	ND2	G5147A	Syn	1	0
142	5153		*ND2*	ND2	A5153G	Syn	0	2
143	5178	rs28357984	*ND2*	ND2	C5178A	Leu→Met	8	5
144	5201		*ND2*	ND2	T5201C	Syn	0	1
145	5205		*ND2*	ND2	T5205C	Syn	0	1
146	5232		*ND2*	ND2	C5232A	Pro→Thr	2	1
147	5237		*ND2*	ND2	G5237A	Syn	1	0
148	5263	rs41320049	*ND2*	ND2	C5263T	Ala→Val	1	2
149	5267		*ND2*	ND2	T5267C	Syn	0	1
150	5291		*ND2*	ND2	T5291C	Syn	1	0
151	5301	rs199794187	*ND2*	ND2	A5301G	Ile→Val	2	2
152	5302		*ND2*	ND2	T5302C	Ile→Thr	0	4
153	5319	rs28456039	*ND2*	ND2	A5319G	Thr→Ala	2	0
154	5351		*ND2*	ND2	A5351G	Syn	1	5
155	5355		*ND2*	ND2	C5355T	Syn	4	4
156	5417		*ND2*	ND2	G5417A	Syn	1	2
157	5442	rs3020601	*ND2*	ND2	T5442C	Phe→Leu	4	1
158	5460	rs3021088	*ND2*	ND2	G5460A	Ala→Thr	1	3
159	5465	rs3902405	*ND2*	ND2	T5465C	Syn	1	0
160	5466		*ND2*	ND2	A5466G	Thr→Ala	1	0
161	5514		TRNW	tRNA-Trp	A5514G		0	1
162	5528		TRNW	tRNA-Trp	T5528C		0	1
163	5563		TRNW	tRNA-Trp	G5563A		1	0
164	5581			Intron	A5581G		1	0
165	5585			Intron	G5585A		0	1
166	5587		TRNA	tRNA-Ala*	T5587C		0	1
167	5628		TRNA	tRNA-Ala*	T5628C		0	1
168	5775		TRNC	tRNA-Cys*	T5775C		1	0
169	5802		TRNC	tRNA-Cys*	T5802C		1	0
170	5821	rs200587831	TRNC	tRNA-Cys*	G5821A		0	1
171	5894			Intron	A5894G		0	12
172	5902			Intron	T5902C		1	0
173	5913	rs201617272	*COX1*	COX1	G5913A	Asp→Asn	0	1
174	5964		*COX1*	COX1	T5964C	Syn	1	0
175	5978		*COX1*	COX1	A5978G	Syn	1	1
176	5987		*COX1*	COX1	A5987G	Syn	1	1
177	6023		*COX1*	COX1	G6023A	Syn	1	0
178	6113		*COX1*	COX1	A6113G	Syn	1	0
179	6179		*COX1*	COX1	G6179A	Syn	2	1
180	6191		*COX1*	COX1	C6191T	Syn	0	1

181	6216		*COX1*	COX1	T6216C	Syn	1	1
182	6260	rs201395766	*COX1*	COX1	G6260A	Syn	1	0
183	6272		*COX1*	COX1	A6272G	Syn	0	1
184	6338		*COX1*	COX1	A6338G	Syn	0	1
185	6351		*COX1*	COX1	T6351C	Syn	1	0
186	6392		*COX1*	COX1	T6392C	Syn	10	7
187	6394		*COX1*	COX1	T6394C	Syn	0	1
188	6413	rs28665937	*COX1*	COX1	T6413C	Syn	1	0
189	6437		*COX1*	COX1	A6437G	Syn	0	1
190	6446		*COX1*	COX1	G6446A	Syn	0	1
191	6452		*COX1*	COX1	C6452T	Syn	0	1
192	6455	rs28516468	*COX1*	COX1	C6455T	Syn	5	5
193	6599		*COX1*	COX1	A6599G	Syn	0	1
194	6614		*COX1*	COX1	T6614C	Syn	1	0
195	6620		*COX1*	COX1	T6620C	Syn	0	1
196	6653		*COX1*	COX1	C6653T	Syn	0	1
197	6680		*COX1*	COX1	T6680C	Syn	1	3
198	6722		*COX1*	COX1	G6722A	Syn	0	1
199	6734	rs41413745	*COX1*	COX1	G6734A	Syn	0	1
200	6752		*COX1*	COX1	A6752G	Syn	0	1
201	6836		*COX1*	COX1	C6836T	Syn	1	0
202	6863		*COX1*	COX1	A6863G	Syn	0	1
203	6908		*COX1*	COX1	T6908C	Syn	1	0
204	6951		*COX1*	COX1	G6951A	Val→Met	1	0
205	6960		*COX1*	COX1	C6960T	Syn	0	3
206	6962	rs1970771	*COX1*	COX1	G6962A	Syn	7	2
207	7055	rs1978002	*COX1*	COX1	A7055G	Syn	1	0
208	7158		*COX1*	COX1	A7158G	Ile→Val	0	2
209	7196	rs28358875	*COX1*	COX1	C7196A	Syn	2	3
210	7250		*COX1*	COX1	A7250G	Syn	2	0
211	7325		*COX1*	COX1	A7325G	Syn	1	0
212	7598		*COX2*	COX2	G7598A	Ala→Thr	2	0
213	7609		*COX2*	COX2	T7609C	Syn	0	1
214	7674		*COX2*	COX2	T7674C	Ile→Thr	0	1
215	7684		*COX2*	COX2	T7684C	Syn	2	4
216	7775		*COX2*	COX2	G7775A	Val→Ile	0	1
217	7828		*COX2*	COX2	A7828G	Syn	2	2
218	7831		*COX2*	COX2	C7831T	Syn	1	0
219	7849		*COX2*	COX2	C7849T	Syn	1	1
220	7852		*COX2*	COX2	G7852A	Syn	0	1!
221	7853	rs199751156	*COX2*	COX2	G7853A	Val→Ile	2	5
222	7861		*COX2*	COX2	T7861C	Syn	2	1
223	7906		*COX2*	COX2	C7906T	Syn	0	1
224	7912		*COX2*	COX2	G7912A	Syn	1	0
225	7961	rs199751156	*COX2*	COX2	T7961C	Syn	1	0
226	7993		*COX2*	COX2	T7993C	Syn	1	0
227	8014		*COX2*	COX2	A8014G	Syn	0	1
228	8020		*COX2*	COX2	G8020A	Syn	1	1
229	8110		*COX2*	COX2	T8110C	Syn	0	2
230	8149		*COX2*	COX2	A8149G	Syn	1	0
231	8260		*COX2*	COX2	T8260C	Syn	1	1
232	8412		*ATP8*	ATP8	T8412C	Met→Thr	0	2
233	8414	rs28358884	*ATP8*	ATP8	C8414T	Leu→Phe	6	3
234	8440		*ATP8*	ATP8	A8440G	Syn	1	0
235	8473	rs371116290	*ATP8*	ATP8	T8473C	Syn	4	0
236	8530		*ATP6*	ATP6	A8530G	Asn→Asp	0	2
237	8563		*ATP6*	ATP6	A8563G	Syn	1	2
238	8575		*ATP6*	ATP6	C8575T	Syn	0	1
239	8584	rs3135028	*ATP6*	ATP6	G8584A	Ala→Thr	2	8
240	8603		*ATP6*	ATP6	T8603C	Phe→Ser	0	1
241	8609		*ATP6*	ATP6	C8609T	Pro→Leu	1	0
242	8654	rs200811540	*ATP6*	ATP6	T8654C	Ile→Thr	0	1
243	8683		*ATP6*	ATP6	A8683G	Thr→Ala	1	0
244	8684	rs201336180	*ATP6*	ATP6	C8684T	Thr→Ser	2	1

245	8701	rs2000975	*ATP6*	ATP6	A8701G	Thr→Ala	19	15
246	8718		*ATP6*	ATP6	A8718G	Syn	1	0
247	8784		*ATP6*	ATP6	A8784G	Syn	0	1
248	8793		*ATP6*	ATP6	C8793T	Syn	2	0
249	8794	rs2298007	*ATP6*	ATP6	C8794T	His→Tyr	1	4
250	8829	rs2000976	*ATP6*	ATP6	C8829T	Syn	0	2
251	8856		*ATP6*	ATP6	G8856A	Syn	2	0
252	8928		*ATP6*	ATP6	T8928C	Syn	0	1
253	8964		*ATP6*	ATP6	C8964T	Syn	1	1
254	8999		*ATP6*	ATP6	T8999C	Val→Ala	1	0
255	9033		*ATP6*	ATP6	A9033G	Syn	1	1
256	9039		*ATP6*	ATP6	G9039A	Syn	3	0
257	9053	rs199646902	*ATP6*	ATP6	G9053A	Ser→Asp	5	1
258	9080		*ATP6*	ATP6	A9080G	Syn	1	0
259	9084		*ATP6*	ATP6	T9084C	Syn	0	3
260	9090		*ATP6*	ATP6	T9090C	Syn	0	1
261	9123	rs28358270	*ATP6*	ATP6	G9123A	Syn	1	0
262	9126		*ATP6*	ATP6	T9126C	Syn	0	1
263	9128	rs199732761	*ATP6*	ATP6	T9128C	Ile→Thr	1	1
264	9180	rs2298011	*ATP6*	ATP6	A9180G	Syn	2	2
265	9242		*COX3*	COX3	A9242G	Syn	0	1
266	9248		*COX3*	COX3	C9248T	Syn	1	0
267	9296		*COX3*	COX3	C9296T	Syn	1	1
268	9449		*COX3*	COX3	C9449T	Syn	0	1
269	9468		*COX3*	COX3	A9468G	Thr→Ala	2	0
270	9530		*COX3*	COX3	T9530C	Syn	0	1
271	9536		*COX3*	COX3	C9536T	Syn	1	1
272	9540	rs2248727	*COX3*	COX3	T9540C	Syn	19	15
273	9545		*COX3*	COX3	A9545G	Syn	1	1
274	9548		*COX3*	COX3	G9548A	Syn	2	0
275	9602		*COX3*	COX3	A9602G	Syn	1	0
276	9682	rs199750417	*COX3*	COX3	T9682C	Thr→Met	1	0
277	9824	rs28411821	*COX3*	COX3	T9824C	Syn	6	6
278	9845		*COX3*	COX3	T9845C	Syn	0	0
279	9856		*COX3*	COX3	T9856C	Ile→Thr	0	1
280	9861		*COX3*	COX3	T9861C	Phe→Leu	1	0
281	9887		*COX3*	COX3	T9887C	Syn	0	1
282	9950	rs3134801	*COX3*	COX3	T9950C	Syn	0	5
283	10029		*TRNG*	tRNA-Gly	A10029G		0	1
284	10031	rs200048690	*TRNG*	tRNA-Gly	T10031C		1	0
285	10034		*TRNG*	tRNA-Gly	T10034C		1	0
286	10097		*ND3*	ND3	A10097C	Syn	0	1
287	10166		*ND3*	ND3	T10166C	Syn	0	1
288	10208		*ND3*	ND3	T10208C	Syn	0	1
289	10238	rs28358275	*ND3*	ND3	T10238C	Syn	1	0
290	10310	rs41467651	*ND3*	ND3	G10310A	Syn	10	8
291	10320	rs193302928	*ND3*	ND3	G10320A	Val→Ile	0	1
292	10345	rs201397417	*ND3*	ND3	C10345T	Ile→Thr	0	1
293	10373	rs28358277	*ND3*	ND3	G10373A	Syn	1	0
294	10397		*ND3*	ND3	A10397G	Syn	2	2
295	10398	rs2853826	*ND3*	ND3	A10398G	Thr→Ala	19	20
296	10400	rs28358278	*ND3*	ND3	C10400T	Syn	19	16
297	10454		TRNR	tRNA-Arg	T10454C		0	1
298	10479		*ND4L*	ND4L	A10479G	Ile→Val	0	1
299	10490		*ND4L*	ND4L	T10490C	Syn	0	1
300	10497		*ND4L*	ND4L	C10497T	Syn	1	0
301	10535		*ND4L*	ND4L	T10535C	Syn	2	1
302	10586	rs28358281	*ND4L*	ND4L	G10586A	Syn	2	2
303	10604		*ND4L*	ND4L	T10604C	Syn	1	0
304	10609	rs200487531	*ND4L*	ND4L	T10609C	Met→Thr	7	2
305	10646		*ND4L*	ND4L	G10646A	Syn	3	0
306	10667		*ND4L*	ND4L	T10667C	Syn	0	1
307	10736		*ND4L*	ND4L	C10736T	Syn	0	1
308	10754		*ND4L*	ND4L	A10754G	Syn	0	1

309	10873	rs2857284	*ND4*	ND4	T10873C	Syn	19	15
310	10897		*ND4*	ND4	C10897T	Syn	0	1
311	10915	rs2857285	*ND4*	ND4	T10915C	Syn	1	1
312	11002		*ND4*	ND4	A11002G	Syn	1	0
313	11016		*ND4*	ND4	G11016A	Ser→Asn	0	1
314	11023		*ND4*	ND4	A11023G	Syn	0	2
315	11038		*ND4*	ND4	A11038G	Syn	1	1
316	11065		*ND4*	ND4	A11065G	Syn	0	1
317	11087	rs28433448	*ND4*	ND4	T11087C	Phe→Leu	0	1
318	11147		*ND4*	ND4	T11147C	Syn	0	1
319	11172	rs2853489	*ND4*	ND4	A11172G	Asn→Ser	0	1
320	11176		*ND4*	ND4	G11176A	Syn	1	0
321	11215		*ND4*	ND4	C11215T	Syn	1	1
322	11236		*ND4*	ND4	C11236T	Syn	0	1
323	11239		*ND4*	ND4	A11239G	Syn	0	1
324	11318		*ND4*	ND4	T11318C	Ser→Pro	1	0
325	11383		*ND4*	ND4	T11383C	Syn	1	0
326	11440		*ND4*	ND4	G11440A	Syn	1	0
327	11465		*ND4*	ND4	T11465C	Syn	0	1
328	11503		*ND4*	ND4	C11503T	Syn	0	1
329	11536		*ND4*	ND4	C11536T	Syn	1	2
330	11632		*ND4*	ND4	C11632T	Syn	1	0
331	11665	rs28631764	*ND4*	ND4	C11665T	Syn	4	2
332	11809		*ND4*	ND4	T11809C	Syn	0	1
333	11878		*ND4*	ND4	T11878C	Syn	0	1
334	11893		*ND4*	ND4	A11893G	Syn	1	0
335	11914	rs2853496	*ND4*	ND4	G11914A	Syn	0	1
336	11918		*ND4*	ND4	T11918G	Ser→Ala	1	0
337	11923		*ND4*	ND4	A11923G	Syn	1	0
338	11944		*ND4*	ND4	T11944C	Syn	2	0
339	12026	rs202136725	*ND4*	ND4	A12026G	Ile→Val	2	0
340	12082	rs372164720	*ND4*	ND4	A12082G	Syn	0	1
341	12091	rs28415973	*ND4*	ND4	T12091C	Syn	4	2
342	12121		*ND4*	ND4	T12121C	Syn	1	0
343	12136		*ND4*	ND4	T12136C	Syn	2	0
344	12153		*TRNH*	tRNA-His	C12153T		1	1
345	12172		*TRNH*	tRNA-His	A12172G		0	1
346	12192	rs3134560	*TRNH*	tRNA-His	G12192A		0	1
347	12224		TRNS2	tRNA-Ser	C12224T		0	2
348	12237		TRNS2	tRNA-Ser	C12237T		1	0
349	12248	rs202114991	TRNS2	tRNA-Ser	A12248G		0	1
350	12250		TRNS2	tRNA-Ser	C12250T		0	1
351	12284		TRNL2	tRNA-Leu	C12284T		1	0
352	12338	rs201863060	*ND5*	ND5	T12338C	Met→Thr	2	1
353	12354		*ND5*	ND5	T12354C	Syn	1	0
354	12358	rs201027657	*ND5*	ND5	A12358G	Thr→Ala	1	2
355	12361	rs3134561	*ND5*	ND5	A12361G	Thr→Ala	0	2
356	12372	rs2853499	*ND5*	ND5	G12372A	Syn	1	2
357	12396		*ND5*	ND5	T12396C	Syn	1	1
358	12405		*ND5*	ND5	C12405T	Syn	0	3
359	12406	rs28617389	*ND5*	ND5	G12406A	Val→Ile	6	3
360	12408		*ND5*	ND5	T12408C	Syn	1	1
361	12501		*ND5*	ND5	G12501C	Met→Ile	1	0
362	12534		*ND5*	ND5	A12534G	Syn	2	0
363	12549		*ND5*	ND5	C12549T	Syn	2	0
364	12609	rs367601393	*ND5*	ND5	T12609C	Syn	1	0
365	12618		*ND5*	ND5	G12618A	Syn	1	0
366	12621		*ND5*	ND5	C12621T	Syn	0	1
367	12630	rs41445245	*ND5*	ND5	G12630A	Syn	2	2
368	12654		*ND5*	ND5	A12654G	Syn	0	1
369	12705		*ND5*	ND5	C12705T	Syn	21	21
370	12714		*ND5*	ND5	T12714C	Syn	1	2
371	12811	rs199974018	*ND5*	ND5	T12811C	Tyr→His	0	3
372	12853	rs28689615	*ND5*	ND5	C12853T	Syn	0	1

373	12882		*ND5*	ND5	C12882T	Syn	7	2
374	12892		*ND5*	ND5	T12892C	Syn	1	0
375	12952		*ND5*	ND5	G12952A	Ala→Thr	1	0
376	12957		*ND5*	ND5	T12957C	Syn	0	1
377	13044		*ND5*	ND5	C13044T	Syn	1	0
378	13104	rs193302960	*ND5*	ND5	A13104G	Syn	1	0
379	13135	rs200044200	*ND5*	ND5	G13135A	Ala→Thr	2	0
380	13145		*ND5*	ND5	G13145A	Ser→Asn	0	1
381	13152		*ND5*	ND5	A13152G	Syn	2	0
382	13182		*ND5*	ND5	T13182C	Syn	1	0
383	13191		*ND5*	ND5	T13191C	Syn	1	0
384	13194		*ND5*	ND5	G13194A	Syn	1	0
385	13215		*ND5*	ND5	T13215C	Syn	1	0
386	13263	rs28359175	*ND5*	ND5	A13263G	Syn	0	1
387	13269	rs28604589	*ND5*	ND5	A13269G	Syn	0	2
388	13350		*ND5*	ND5	A13350G	Syn	1	0
389	13395		*ND5*	ND5	A13395G	Syn	1	1
390	13422		*ND5*	ND5	A13422G	Syn	1	0
391	13434		*ND5*	ND5	A13434G	Syn	2	0
392	13500		*ND5*	ND5	T13500C	Syn	1	0
393	13563		*ND5*	ND5	A13563G	Syn	1	0
394	13590	rs28359177	*ND5*	ND5	G13590A	Syn	1	0
395	13602		*ND5*	ND5	T13602C	Syn	1	1
396	13626		*ND5*	ND5	C13626T	Syn	1	0
397	13650	rs2854123	*ND5*	ND5	C13650T	Syn	1	2
398	13707	rs193302965	*ND5*	ND5	G13707A	Syn	0	2
399	13708	rs28359178	*ND5*	ND5	G13708A	Ala→Thr	2	3
400	13759		*ND5*	ND5	G13759A	Ala→Thr	7	1
401	13834		*ND5*	ND5	A13834G	Thr→Ala	1	0
402	13928	rs28359184	*ND5*	ND5	G13928C	Ser→Thr	13	9
403	13930		*ND5*	ND5	A13930G	Ile→Val	0	1
404	13953		*ND5*	ND5	T13953C	Syn	1	0
405	13966	rs41535848	*ND5*	ND5	A13966G	Thr→Ala	1	0
406	13980		*ND5*	ND5	G13980A	Syn	0	2
407	14053		*ND5*	ND5	A14053G	Thr→Ala	0	1
408	14063		*ND5*	ND5	T14063C	Ile→Thr	1	0
409	14115		*ND5*	ND5	C14115T	Syn	1	0
410	14129	rs386829201	*ND5*	ND5	C14129T	Thr→Ile	1	0
411	14148		*ND5*	ND5	A14148G	Syn	0	1
412	14178	rs28357671	*ND6*	ND6	T14178C	Asn→Lys	0	1
413	14180	rs200933339	*ND6*	ND6	A14180G	Thr→Cys	0	1
414	14302		*ND6*	ND6	T14302C	Ser→Pro	0	1
415	14308	rs28357674	*ND6*	ND6	T14308C	Ser→Pro	1	2
416	14318	rs28357675	*ND6*	ND6	A14318G	Asn→Ser	0	1
417	14388		*ND6*	ND6	A14388G	Syn	0	5
418	14392		*ND6*	ND6	C14392T	His→Tyr	1	0
419	14470		*ND6*	ND6	T14470C	Ser→Pro	2	1
420	14502	rs201327354	*ND6*	ND6	A14502G	Ile→Val	2	0
421	14554		*ND6*	ND6	A14554G	Syn	0	1
422	14632		*ND6*	ND6	C14632T	Syn	0	1
423	14668	rs28357678	*ND6*	ND6	C14668T	His→Tyr	6	3
424	14766	rs527236041	*CYTB*	CYTB	T14766C	Ile→Thr	33	34
425	14783	rs527236042	*CYTB*	CYTB	T14783C	Syn	17	13
426	14953		*CYTB*	CYTB	C14953T	Syn	1	0
427	14971		*CYTB*	CYTB	T14971C	Syn	0	1
428	14978	rs199997767	*CYTB*	CYTB	A14978G	Ile→Val	0	1
429	14979	rs200786872	*CYTB*	CYTB	T14979C	Ile→Thr	3	0
430	14989		*CYTB*	CYTB	C14989T	Syn	0	1
431	15024		*CYTB*	CYTB	G15024A	Cys→Tyr	0	1
432	15038	rs202045169	*CYTB*	CYTB	A15038G	Ile→Val	1	0
433	15040		*CYTB*	CYTB	C15040T	Syn	2	0
434	15043	rs527236043	*CYTB*	CYTB	G15043A	Syn	19	16
435	15066		*CYTB*	CYTB	T15066A	Phe→Tyr	1	0
436	15071	rs199999794	*CYTB*	CYTB	T15071C	Tyr→His	2	0

437	15172		*CYTB*	CYTB	G15172A	Syn	1	0
438	15178		*CYTB*	CYTB	A15178G	Syn	1	0
439	15209		*CYTB*	CYTB	T15209C	Tyr→His	0	1
440	15211		*CYTB*	CYTB	C15211T	Syn	1	0
441	15217		*CYTB*	CYTB	G15217A	Syn	0	2
442	15218	rs2853506	*CYTB*	CYTB	A15218G	Thr→Ala	2	0
443	15220		*CYTB*	CYTB	A15220G	Syn	1	0
444	15223		*CYTB*	CYTB	C15223T	Syn	0	2
445	15226	rs527236174	*CYTB*	CYTB	A15226G	Syn	0	1
446	15235		*CYTB*	CYTB	A15235G	Syn	0	3
447	15236	rs201250154	*CYTB*	CYTB	A15236G	Ile→Val	3	1
448	15241		*CYTB*	CYTB	A05241G	Syn	0	1
449	15301	rs527236045	*CYTB*	CYTB	G15301A	Syn	20	15
450	15313		*CYTB*	CYTB	T15313C	Syn	0	1
451	15338		*CYTB*	CYTB	C15338T	Syn	0	1
452	15340		*CYTB*	CYTB	A15340G	Syn	1	0
453	15346	rs527236180	*CYTB*	CYTB	G15346A	Syn	2	0
454	15355		*CYTB*	CYTB	G15355A	Syn	1	0
455	15412		*CYTB*	CYTB	T15412G	Syn	0	1
456	15440		*CYTB*	CYTB	T15440C	Syn	0	1
457	15442		*CYTB*	CYTB	A15442G	Syn	1	1
458	15479		*CYTB*	CYTB	T15479C	Phe→Leu	1	0
459	15481		*CYTB*	CYTB	C15481T	Syn	1	0
460	15487	rs28357370	*CYTB*	CYTB	A15487T	Syn	2	3
461	15508		*CYTB*	CYTB	C15508T	Syn	0	2
462	15535	rs28357371	*CYTB*	CYTB	C15535T	Syn	1	0
463	15565		*CYTB*	CYTB	T15565C	Syn	1	1
464	15643		*CYTB*	CYTB	C15643T	Syn	1	0
465	15662	rs3094280	*CYTB*	CYTB	A15662G	Ile→Val	0	2
466	15670	rs527236211	*CYTB*	CYTB	T15670C	Syn	1	2
467	15724		*CYTB*	CYTB	A15724G	Syn	0	2
468	15784	rs527236194	*CYTB*	CYTB	T15784C	Syn	1	1
469	15787		*CYTB*	CYTB	T15787C	Syn	1	0
470	15832	rs193302999	*CYTB*	CYTB	C15832T	Syn	1	0
471	15850		*CYTB*	CYTB	T15850C	Syn	0	1
472	15851	rs3094281	*CYTB*	CYTB	A15851G	Syn	0	1
473	15884	rs527236195	*CYTB*	CYTB	G15884A	Syn	0	1
474	15889	rs199833246	*TRNT*	tRNA-Thr	T15889C		1	0
475	15924	rs2853510	*TRNT*	tRNA-Thr	A15924G		1	0
476	15927	rs193303002	*TRNT*	tRNA-Thr	G15924A		0	1
477	15930	rs41441949	*TRNT*	tRNA-Thr	G15930A		0	1
478	16041	rs369904200		D-loop	A16041G		1	0
479	16051	rs117565943		D-loop	A16051G		0	1
480	16086			D-loop	T16086C		1	0
481	16092			D-loop	T16092C		5	1
482	16093	rs2853511		D-loop	C16093T		1	2
483	16108			D-loop	C16108T		1	0
484	16111	rs35315169		D-loop	C16111T		0	3
485	16126	rs147029798		D-loop	T16126C		1	3
486	16129	rs41534744		D-loop	G16129A		12	7
487	16136	T16136C		D-loop	T16136C		1	0
488	16140	rs3134562		D-loop	T16140C		2	2
489	16145	rs41419246		D-loop	G16145A		0	1
490	16150			D-loop	C16150T		1	0
491	16158	rs386829276		D-loop	A16158G		0	1
492	16162	rs41466049		D-loop	A16162G		1	0
493	16163	rs41479950		D-loop	A16163G		0	1
494	16164			D-loop	A16164G		2	0
495	16166			D-loop	A16166G		1	0
496	16168	rs371419667		D-loop	C16168T		1	0
497	16171			D-loop	A16171G		0	1
498	16172	rs2853817		D-loop	T16172C		8	2
499	16181			D-loop	A16181G		0	1
500	16182			D-loop	A16182C		0	6

501	16183	rs34100702	D-loop	A16183C	3	17
502	16184		D-loop	C16184T	1	1
503	16185		D-loop	C16185T	0	1
504	16189	rs28693675	D-loop	T16189C	6	11
505	16192		D-loop	C16192T	0	3
506	16194		D-loop	A16194C	0	1
507	16195		D-loop	T16195C	0	1
508	16203		D-loop	A16203G	0	1
509	16207		D-loop	A16207G	1	1
510	16217	rs35134837	D-loop	T16217C	3	2
511	16223	rs2853513	D-loop	C16223T	16	19
512	16234	rs368259300	D-loop	C16234T	1	4
513	16235		D-loop	A16235G	1	2
514	16242	rs386829288	D-loop	C16242T	1	1
515	16243		D-loop	C16243T	0	1
516	16245	rs386829289	D-loop	C16245T	0	1
517	16248	rs386829290	D-loop	C16248T	0	1
518	16249	rs372301309	D-loop	T16249C	1	0
519	16257		D-loop	C16257A	0	2
520	16259		D-loop	C16259T	1	0
521	16260		D-loop	C16260T	1	2
522	16261	rs138126107	D-loop	C16261T	1	5
523	16263		D-loop	T16263C	1	1
524	16265		D-loop	A16265C	0	1
525	16266		D-loop	C16266A	0	4
526	16270	rs2857290	D-loop	C16270T	1	0
527	16274	rs144095641	D-loop	G16274A	1	0
528	16278	rs41458645	D-loop	C16278T	1	0
529	16288		D-loop	T16288C	1	1
530	16290	rs386829301	D-loop	C16290T	1	4
531	16291	rs35302802	D-loop	C16291T	2	3
532	16293		D-loop	A16293T	2	0
533	16295		D-loop	C16295T	4	1
534	16297		D-loop	T16297C	0	3
525	16298	rs148377232	D-loop	T16298C	2	6
536	16304		D-loop	T16304C	12	7
537	16309	rs373517769	D-loop	A16309G	1	2
538	16311	rs34799580	D-loop	T16311C	6	3
539	16316		D-loop	A16316G	1	3
540	16318		D-loop	A16318T	0	1
541	16319	rs35105996	D-loop	G16319A	3	6
542	16327	rs41355449	D-loop	C16327T	0	1
543	16335		D-loop	A16335G	1	1
544	16354		D-loop	C16354T	1	0
545	16355	rs138576863	D-loop	C16355T	0	1
546	16356	rs386829310	D-loop	T16356C	0	1
547	16359	rs370567324	D-loop	T16359C	1	0
548	16360	rs117017250	D-loop	C16360T	1	1
549	16362	rs62581341	D-loop	T16362C	12	10
550	16390	rs41378955	D-loop	G16390A	3	2
551	16399	rs139001869	D-loop	A16399G	1	1
552	16463		D-loop	A16463G	0	1
553	16468		D-loop	T16468C	1	0
554	16470		D-loop	G16470A	1	0
555	16471		D-loop	G16471A	1	0
556	16473		D-loop	G16473A	1	0
557	16497		D-loop	A16497G	1	1
558	16519	rs3937033	D-loop	T16519C	22	19
	Total				972	1013

Abbreviation: Syn, synonymous. * light strand.

Table S5. Ratio of nonsynonymous (NS) to synonymous substitutions in mtDNA genes in tuberculosis patients with and without drug-induced liver injury (DILI).

mtDNA gene	Ratio of NS/synonymous substitutions		*p*
	DILI (n=38)	No DILI (n=38)	
**ND1 (NADH dehydrogenase subunit 1)**	0.42 (16/38)	0.53 (16/30)	0.737
ND2 (NADH dehydrogenase subunit 2)	0.35 (23/66)	0.68 (27/40)	0.082
COX1 (Cytochrome c oxidase subunit 1)	0.02 (1/43)	0.10 (4/40)	0.357
COX2 (Cytochrome c oxidase subunit 2)	0.29 (4/14)	0.44 (7/16)	0.815
ATP8 (ATP synthase F0 subunit 8)	1.20 (6/5)	∞ (5/0)	0.216
ATP6 (ATP synthase F0 subunit 6)	3.36 (37/11)	2.13 (34/16)	0.435
COX3 (Cytochrome c oxidase subunit 3)	0.13 (4/32)	0.03 (1/33)	0.389
ND3 (NADH dehydrogenase subunit 3)	0.58 (19/33)	0.76 (22/29)	0.629
ND4L (NADH dehydrogenase subunit 4L)	0.45 (5/11)	0.43 (3/7)	0.712
ND4 (NADH dehydrogenase subunit 4)	0.09 (4/43)	0.08 (3/37)	0.824
ND5 (NADH dehydrogenase subunit 5)	0.62 (39/63)	0.48 (25/52)	0.522
ND6 (NADH dehydrogenase subunit 6)	1.40 (7/5)	1.13 (9/8)	0.927
CYTB (Cytochrome b)	0.28 (27/97)	0.23 (21/93)	0.630
Overall	0.25 (192/780)	0.21 (177/836)	0.212

Journal of
Clinical Medicine

MDPI

Table S6. Number of haplogroup-associated polymorphisms found in tuberculosis patients with drug-induced liver injury (DILI) (n = 38) and without DILI (n = 38).

Mitochondrial region (gene)	No. of haplogroup-associated polymorphisms
D-loop	1
RRNS	4
RRNL	4
ND1	4
ND2	8
Intron	1
COX1	6
COX2	1
ATP8	2
ATP6	1
COX3	5
ND3	4
ND4L	1
ND4	5
ND5	9
ND6	2
CYTB	10
Total	68
